# Standard technique in Japan for measuring hepatic venous pressure gradient

**DOI:** 10.1007/s00535-024-02182-z

**Published:** 2024-12-09

**Authors:** Yusuke Imai, Yohei Koizumi, Yoichi Hiasa, Masashi Hirooka, Yoshio Tokumoto, Osamu Yoshida, Fumio Chikamori

**Affiliations:** 1 Department of Gastroenterology and Metabology, Ehime University Graduate School of Medicine, Toon City, Ehime 791-0295 Japan; 2 Department of Surgery, Japanese Red Cross Kochi Hospital, Hadaminami-Machi, Kochi City, Kochi 780-8562 Japan

**Keywords:** PVP, HVPG, WHVP, Elastography, Baveno VII

## Abstract

**Background:**

Direct measurement of portal venous pressure (PVP) is invasive, so the hepatic venous pressure gradient (HVPG) is commonly measured to evaluate portal hypertension (PH). HVPG is the gold standard for estimating PVP but few reports have covered standardized measurement techniques.

**Methods:**

This study validated standardized techniques for PVP measurement.

**Results:**

In Western countries, electronic transducers are commonly used to measure PVP, whereas the water column method is still frequently applied in Japan. Setting a reference point for accurate PVP measurement is important but complicated. According to Japanese guidelines, the reference point for PVP measurement is 10 cm above the dorsal surface or in the midaxillary line. For simpler determination, the anterior axillary point, defined as the point of convergence between the proximal pectoralis major muscle and arm when both arms are positioned against the trunk in a supine position, can be used as the reference point. New methods, such as endoscopic ultrasound-guided portal pressure gradient, offer less invasive alternatives. Non-invasive methods like elastography measure liver and spleen stiffness, which correlate with HVPG. The Baveno VII criteria incorporate measurements of liver and splenic stiffness for risk stratification. Biomarkers such as type IV collagen, M2BPGi, and FIB-4 score also predict HVPG. The Baveno VII consensus emphasizes the status of HVPG as the gold standard while advocating for non-invasive alternative methods to improve patient care and monitor treatment efficacy.

**Conclusions:**

Continued development of non-invasive tests is crucial for safer, more convenient PH management.

## Introduction

**Fig. 1:**
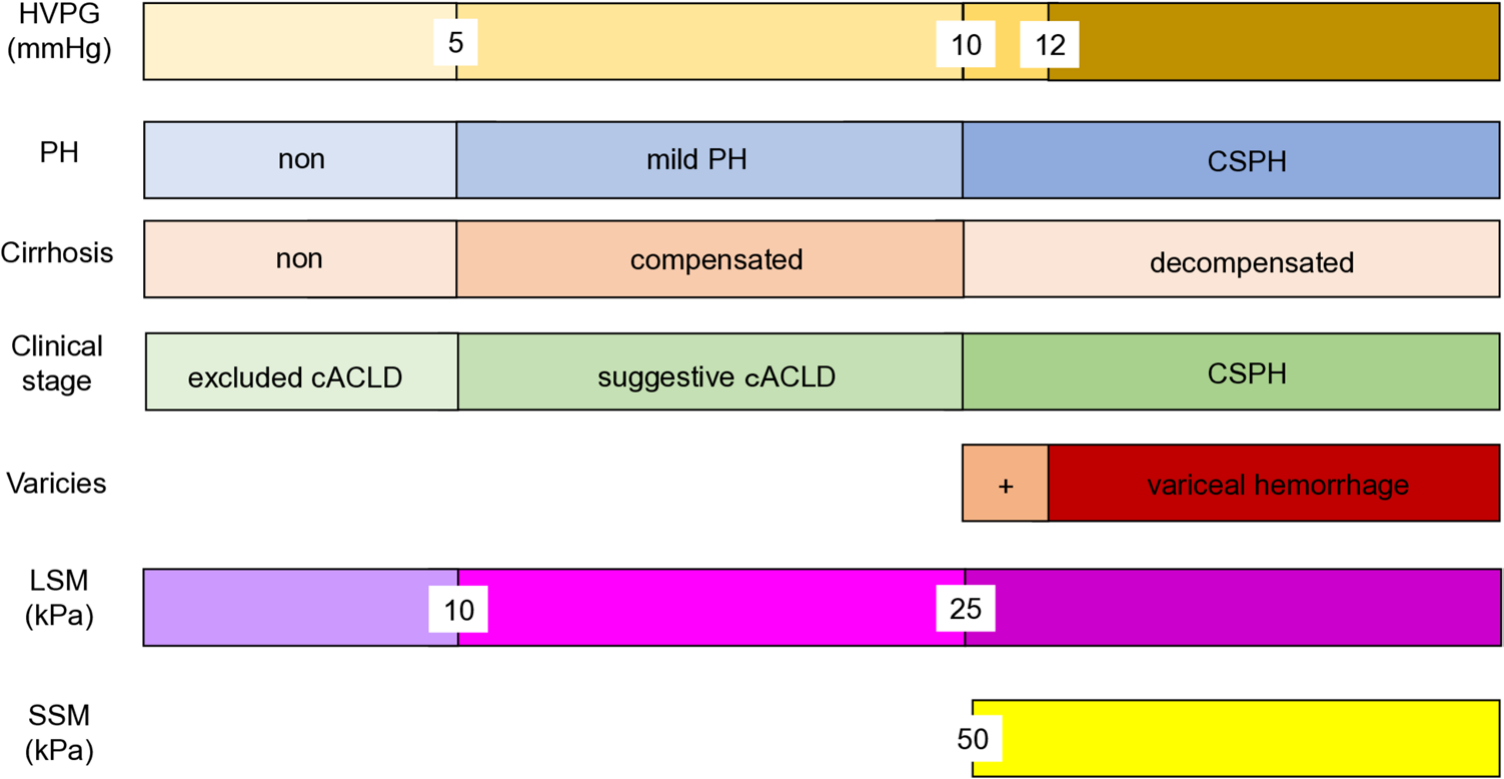
Hepatic venous pressure gradient and portal hypertension. Relationships between HVPG, other noninvasive tests, and clinical manifestations. *cACLD* compensated advanced chronic liver disease, *CSPH* clinically significant portal hypertension, *HVPG* hepatic venous pressure gradient, *LSM* liver stiffness measurement, *PH* portal hypertension, *SSM* splenic stiffness measurement. Modified from the figures of De Franchis et al. [5], Suk [17], Garcia-Tsao et al. [6], and Albilllos et al. [7]

Direct measurement of portal venous pressure (PVP) is invasive and not straightforward. In 1951, Myers and Taylor first reported wedged hepatic venous pressure (WHVP) as an indirect method of measuring PVP [1]. Evaluation of PVP is essential for understanding the pathophysiology and hemodynamics of portal hypertension (PH). As no valves are present in the portal system or splenic vein, increases in blood flow resistance anywhere between the splenic vein and right atrium will lead to increased portal and/or splenic venous pressure, eventually resulting in PH. PH is classified into three types based on the site of increased blood flow resistance: prehepatic, intrahepatic, and posthepatic [2]. Cirrhosis results in intrahepatic PH and is the most common cause of PH [3, 4]. PH is a central underlying pathology for complications seen in patients with chronic liver disease or cirrhosis, and accurate diagnosis of PH holds significant clinical importance for considerations of prognosis. HVPG ≥ 5 mmHg indicates PH, and ≥ 10 mmHg represents clinically significant PH (CSPH) [5–7]. HVPG ≥ 12 mmHg carries a risk of progression to decompensated cirrhosis and variceal bleeding (Fig. 1) [6, 7].

**Fig. 2:**
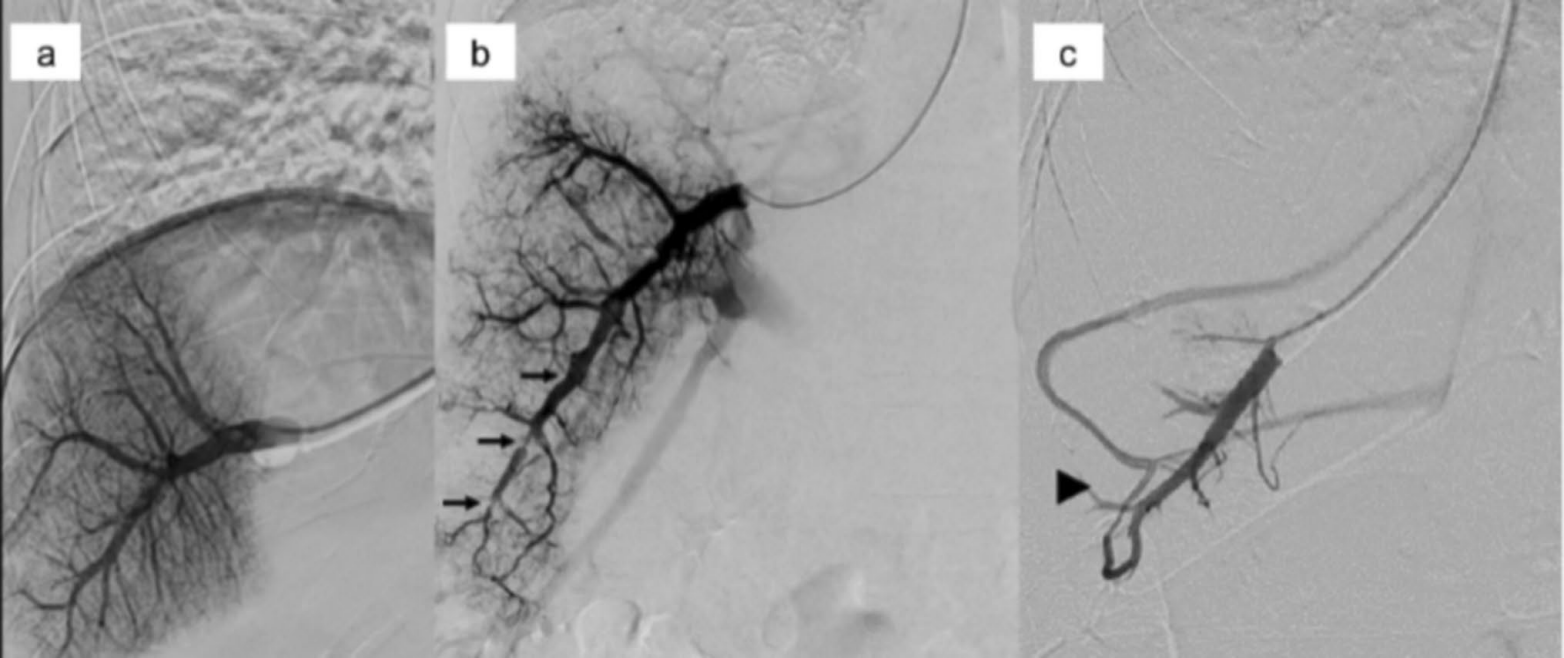
Hepatic venography image during HVPG measurement. **a** Homogeneous findings on sinusoidgram. **b** Inhomogeneous findings on sinusoidgram and irregular findings of the hepatic vein (arrow). **c** Hepatic venous anastomosis (arrowhead)

The current standard for estimating PVP is measurement of HVPG [8–12]. In the Baveno VII consensus, while the necessity for the promotion of non-invasive tests is mentioned, HVPG is considered as the gold standard for diagnosing PH [5]. Furthermore, measuring HVPG can provide more clinical information by performing hepatic venography during catheter insertion, such as irregularities in the hepatic veins suggestive of cirrhosis and the presence of hepatic venous shunts (Fig. 2) [13]. In addition, utility of HVPG is supported by a long history of accumulated evidence, such as its ability to predict the risk of esophageal variceal rupture [6, 7].

Although HVPG measurement is widely practiced, few reports have summarized standard techniques for the devices and methods used to achieve this measurement. This study aimed to provide an overview and validation of standardized examination techniques for measuring portal pressure in Japan.

## Baveno VII consensus and HVPG

In 2021, the Baveno VII consensus meeting was held, announcing new recommendations regarding PH as the Baveno VII consensus. New criteria for compensated advanced chronic liver disease (cACLD), CSPH, individualized management of esophagogastric varices, and other aspects were introduced [14]. Recommendations regarding HVPG were also provided. In the Baveno VII consensus, HVPG remains the gold standard for diagnosing PH. In addition, measurement of HVPG is recommended when assessing the efficacy of treatment for CSPH. The Baveno VII consensus also mentioned potential variations in HVPG values based on underlying liver pathologies such as viral hepatitis and non-alcoholic steatohepatitis, although careful interpretation of measured HVPG is needed [5].

While highlighting the importance of HVPG in diagnosing CSPH, the Baveno VII consensus also discussed the utility of non-invasive tests. The previous Baveno VI consensus proposed risk stratification for CSPH using LSM measured by elastography [15]. The Baveno VII consensus stated that LSM ≤ 15 kPa and platelet count > 150 × 10^9^/dL can be used to rule out CSPH. Further, in cases of patients with virus- and/or alcohol-related and non-obese non-alcoholic steatohepatitis-related cACLD, LSM ≥ 25 kPa is sufficient to identify CSPH. Moreover, the Baveno VII consensus suggested that risk stratification for CSPH could also be achieved using SSM. SSM can be used to rule out and rule in CSPH (SSM < 21 kPa and SSM > 50 kPa, respectively) [5].

## Methods of measuring PVP

### PVP

PVP is considered normal at 100–150 mmH_2_O (7.4–11.0 mmHg), with 1 mmH_2_O equivalent to 0.0736 mmHg and 1 mmHg equivalent to 13.6 mmH_2_O. PH is defined as a constant PVP ≥ 200 mmH_2_O (14.7 mmHg). Direct and indirect methods can be used for measuring portal pressure (Table 1). Devices used for pressure measurement include the water column method and electronic transducers [16]. While the use of electronic transducers is more common in Western countries, many facilities in Japan still utilize the water column due to the lack of a requirement for specialized equipment. The antecubital, right jugular or femoral veins are commonly selected as puncture routes [17]. In Japan, Yamamoto et al. reported that puncture of antecubital veins is less invasive and more convenient in terms of risk of bleeding and postoperative management [18].

**Table 1:** Methods of measuring portal vein pressure

Direct portal pressure measurement
Intraoperative portal pressure measurement
Transhepatic percutaneous portal pressure measurement
Indirect portal pressure measurement
Wedged hepatic venous pressure (WHVP) measurement
Hepatic venous pressure gradient (HVPG) measurement

### Methods for measuring WHVP and HVPG: are WHVP and FHVP being evaluated correctly?

Measurement of WHVP: The procedure involves inserting a balloon catheter into the hepatic vein via the right antecubital, right jugular, or femoral veins and inflating the balloon within the hepatic vein for pressure measurement, or wedging the catheter tip into the hepatic vein to occlude the vessel while measuring pressure [19, 20]. When wedging the catheter tip into the hepatic vein for measurement, it is important to note that the degree of fibrosis varies between different regions of each vein, which may lead to fluctuations in measured values [21]. In addition, if an anastomosis is observed at the distal end of the hepatic vein, portal pressure may be underestimated, so the presence of hepatic vein anastomosis needs to be ruled out by pre-measurement imaging [22].

Measurement of HVPG: HVPG is currently the most commonly used indicator of portal pressure [5]. HVPG is calculated as the difference between WHVP and free hepatic venous pressure (FHVP) [23, 24]. Both WHVP and FHVP are influenced by intra-abdominal pressure and the position of the reference point, whereas HVPG is unaffected by these factors.

### Setting the reference point for PVP measurement

**Fig. 3:**
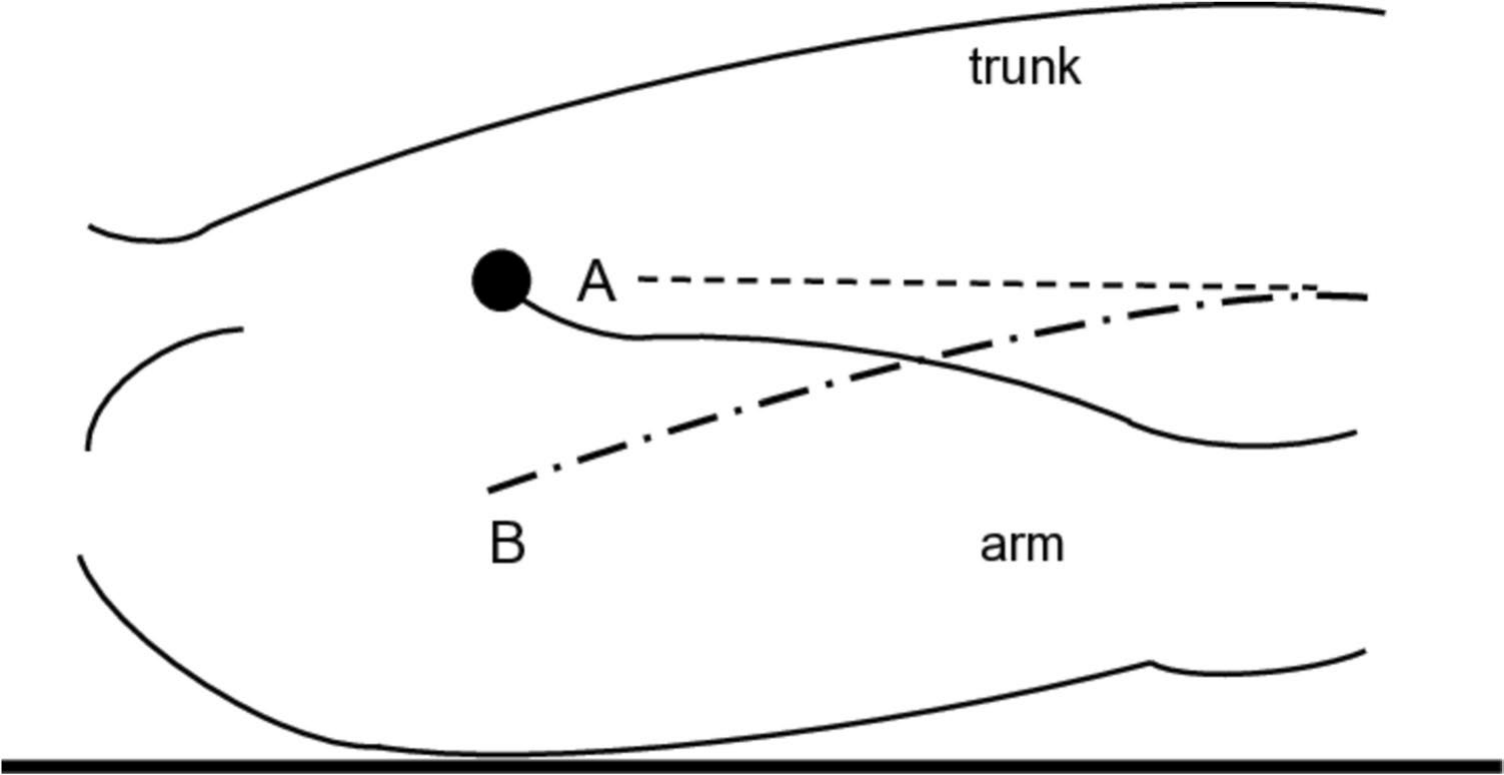
Anterior axillary point as a reference point for PVP measurement. Point A: Anterior axillary point is the convergence point between the proximal pectoralis major and the arm with both arms positioned against the trunk. The height of the anterior axillary point is approximately equal to the height of the midaxillary line (Line B) of the upper abdomen

When measuring portal pressure, the reference point corresponds to the height of the transducer and is conventionally positioned at the level of the right atrium (midaxillary line) [16, 25]. While it is common in central venous pressure (CVP) measurement to use the superior vena cava as the reference point, methods for estimating the position of the superior vena cava from the body surface can be inconsistent. Such inconsistency is likely due to the ambiguity of the axillary line as an imaging reference. Specifically, variations arise depending on whether the axillary line is drawn curved along the trunk axis, leading to differences in height for setting the reference point in the axilla or on the chest or abdomen. According to Japanese guidelines (The General Rules for Study of Portal Hypertension, 4th edition), the reference point for PVP measurement is typically established 10 cm above the back of the patient in the supine position or the midaxillary line. In this guideline, the utility of using CT to measure the height of the portal vein from the back and employing this as the reference point has been suggested. In addition, for simplicity in determining the reference point for a supine individual, the “anterior axillary point” is considered beneficial as the reference point for portal pressure measurement and defined as the convergence point between the proximal pectoralis major and the arm with both arms positioned against the trunk (Fig. 3).

### New methods for measuring portal pressure

HVPG serves as the gold standard for evaluating PH, but poses issues due to its invasive nature. HVPG cannot assess presinusoidal or prehepatic PH and is not useful in the presence of hepatic venous anastomosis (Fig. 2c) [16, 22, 26]. In addition, the diagnostic accuracy of HVPG in metabolic-associated fatty liver disease (MAFLD), a major cause of chronic liver disease, is under scrutiny [27]. Given the widespread use of endoscopic ultrasound (EUS) in routine gastrointestinal practice, this modality may be applicable to methods of PVP measurement. As an alternative to HVPG measurement, a new technique has been reported involving direct puncture of the portal vein under EUS guidance, followed by measurement of portal pressure using a digital pressure gage. Huang et al. established the clinical feasibility of measuring EUS-guided portal pressure gradient (EUS-PPG) and examined its correlation with HVPG [28]. Several cohort studies have validated the safety and feasibility of EUS-PPG in clinical settings [29–31]. In terms of procedural skills, EUS-PPG primarily relies on the technique of fine needle aspiration needles, leveraging an already acquired skill set, and holds potential to overcome most of the aforementioned limitations of HVPG. However, reports correlating EUS-PPG with HVPG simultaneously are scarce [32]. The ongoing ENCOUNTER trial (NCT 04987034) aims to prospectively evaluate the correlation between EUS-PPG and HVPG, and the results are eagerly anticipated.

## Hemodynamics and HVPG

### Cardiac output and HVPG

In PH, the increase in intrahepatic vascular resistance and hyperdynamic circulation of splanchnic blood flow, and the development of the collateral circulation termed “splanchnic caput medusa” by Chikamori et al. lead to alterations in systemic hemodynamics [33, 34]. In a state of PH, the production of nitric oxide from vascular endothelial cells is increased, causing vasodilation and consequent decreases in systemic vascular resistance index [35]. In addition, the increases in stroke volume and heart rate in PH lead to an increase in cardiac index (CI), resulting in a hyperdynamic circulatory state.

CI shows positive correlations with WHVP, HVPG, and Child–Pugh score, and negative correlations with prothrombin time and hepaplastin test [33]. Child–Pugh Grade A shows lower HVPG and cardiac output compared to Child–Pugh Grades B and C [36]. Furthermore, Chikamori et al. reported a significant increase in CI with increasing severity esophageal varices [37].

### Beta-blockers and HVPG

Beta-blockers are used to prevent the rupture of esophageal varices by lowering portal pressure [38, 39]. Non-selective beta-blockers (NSBBs) reduce portal pressure through two main mechanisms: a decrease in cardiac output via beta-1 blockade and reductions in portal blood flow due to the constriction of abdominal visceral blood vessels via beta-2 blockade [40].

Cases in which HVPG decreased by > 10% with NSBB treatment have been observed to show significant reductions in variceal bleeding [41]. In addition, the incidence of esophagogastric varices is also significantly reduced in cases where NSBBs decrease HVPG by > 10% [42]. Measuring HVPG is therefore important to confirm the portal pressure-lowering effects of beta-blockers. However, as frequent measurement of HVPG is difficult, heart rate monitoring is often used as a substitute in practice [12, 43].

Compared to traditional beta-blockers like propranolol, the third-generation beta-blocker carvedilol has been reported to offer superior efficacy in lowering HVPG. Further, carvedilol significantly reduces mortality in cirrhotic patients with esophagogastric varices and shows a lower incidence of adverse events compared to classical beta-blockers [44, 45].

## Non-invasive tests for assessing liver fibrosis and HVPG

### Ultrasound elastography and HVPG

Measurement of HVPG is considered the gold standard for evaluating PH, but has the problem of being invasive. This makes it difficult to perform repeated tests within short periods, such as for monitoring treatment efficacy. Attempts have therefore been made to substitute HVPG with safe and convenient non-invasive tests (Table 2).

**Table 2:** Diagnosis of CSPH by HVPG measurement and other testing methods

Method	Reference value	Study (references)
HVPG	10 mmHg	Baveno VII [5]
LSM (TE)	25 kPa	Baveno VII [5]
SSM (TE)	50 kPa	Baveno VII [5]
SSM (RTE)	8.24	Hirooka et al. [49]
Diameter of cisterna chyli	5.4 mm	Yano et al. [60]
Diameter of tDT	3.7 mm	Yano et al. [60]
M2BPGi	2.33 C.O.I	Wu et al. [71]

*HVPG* hepatic venous pressure gradient, *LSM* liver stiffness measurement, *M2BPGi* Mac-2 binding protein glycosylation isomer, *SSM* splenic stiffness measurement, *tDT* terminal thoracic duct, *TE* transient elastography, *RTE* real-time tissue elastography

Liver biopsy has been considered the gold standard for diagnosing liver fibrosis, but less invasive tests have become more widely used in recent years. Elastography using ultrasound can measure liver and spleen stiffness noninvasively. Two main methods are applied in elastography: one measures the strain distribution when tissue is compressed to visualize the relative stiffness distribution (real-time tissue elastography, etc.), and the other measures the shear wave propagation velocity distribution when tissue is vibrated to visualize the quantitative stiffness distribution (transient elastography, etc.) [46]. Correlations have been shown between liver stiffness as measured by elastography and HVPG [47]. Pons et al. reported that liver stiffness as measured by transient elastography predicts CSPH, defined as HVPG ≥ 10 mmHg, when liver stiffness measurement (LSM) is ≥ 25 kPa [48]. This diagnostic criterion has also been adopted in the Baveno VII criteria [5].

In addition, spleen stiffness reportedly correlates with HVPG [49–52]. Hirooka et al. set a cutoff value of 8.24 for spleen stiffness as measured by real-time tissue elastography to predict HVPG ≥ 10 mmHg, achieving 90% diagnostic accuracy for esophagogastric varices. Splenic stiffness measurement (SSM) as measured by transient elastography has also been reported to correlate with HVPG [53]. The increase in spleen stiffness is attributed to PH, leading to hyperplasia of reticular tissue, elongation of terminal arterioles, enlargement of the white pulp, and fibrosis between trabeculae [49]. SSM correlates with HVPG better than LSM does [49, 54]. Elastography offers the advantages of bedside applicability and real-time information. However, caution is needed as measurement results may be affected by liver inflammation, liver congestion, and dietary intake [55, 56].

### Imaging of the lymphatic vascular system and HVPG

In PH, sinusoidal pressure elevation leads to an approximately 30-fold increase in lymph fluid production by the liver [57]. Lymph fluid produced by the liver flows into the veins through the cisterna chyli and thoracic duct, and changes the shape of these vessels due to changes in portal pressure [58]. Yano et al. evaluated the cisterna chyli and thoracic duct using ultrasound and computed tomography, reporting a positive correlation between HVPG and diameters of the cisterna chyli and thoracic duct. They reported that the diameters of the cisterna chyli (≤ 4.5 mm to exclude CSPH; ≥ 5.4 mm to diagnose CSPH) and terminal thoracic duct (≤ 2.1 mm to exclude CSPH; ≥ 3.7 mm to diagnose CSPH) are useful for diagnosing CSPH. They also noted that during the compensated phase, these vessels tend to narrow, posing a risk for the development of refractory ascites. In addition, by incorporating lymphatic vessel diameter into the Baveno VII criteria, non-invasively and accurately identifying CSPH patients is suggested to become feasible [59].

### Other imaging tests and HVPG

Magnetic resonance elastography (MRE) is a method that uses an external actuator to propagate elastic waves through the liver and measures these with MRI [60]. Reports suggest that MRE has a higher diagnostic accuracy for liver fibrosis than ultrasound elastography [61]. Liver and spleen stiffnesses measured by MRE correlate well with HVPG, with spleen stiffness measured by 3D-MRE showing the best correlation [62, 63].

Subharmonic-aided pressure estimation (SHAPE) is a diagnostic method that utilizes contrast-enhanced ultrasound [64]. The size of microbubbles within blood vessels changes with variations in hydrostatic pressure. SHAPE measures these pressure changes using signals in the subharmonic band, which are most sensitive to these changes in microbubble size [65]. SHAPE has been reported to correlate with HVPG and is also useful for monitoring treatment effects [66, 67].

### Biomarkers of liver fibrosis and HVPG

Attempts have been made to predict HVPG using serum biomarkers of liver fibrosis. Type IV collagen is a major component of the extracellular matrix found in the digestive tract, skin, kidneys, and liver. In chronic liver diseases, levels rise with the progression of liver fibrosis. Levels of type IV collagen are reportedly significantly elevated in cases where HVPG is ≥ 10 mmHg [68].

M2BPGi is a glycosylated isoform of Mac-2 binding protein produced by hepatic cells and has been developed as a novel glycan marker reflecting the progression of liver fibrosis [69, 70]. Wu et al. reported that M2BPGi levels ≥ 2.33 C.O.I. can predict CSPH (HVPG ≥ 10 mmHg) [71].

FIB-4 score and APRI are non-invasive diagnostic scoring systems used to assess liver fibrosis. FIB-4 score is calculated using parameters such as age, aspartate aminotransferase (AST) concentration, alanine aminotransferase (ALT) concentration, and platelet count. APRI is calculated using parameters such as AST, ALT, and platelet count. FIB-4 score and APRI reportedly correlate with HVPG and predict CSPH [72–74].

## Conclusion

Measurement of HVPG remains the gold standard for diagnosing PH and continues to hold a significant position in clinical practice. However, the active development of safer and more convenient non-invasive testing methods that could potentially be substituted for HVPG is expected to continue.

## Abbreviations

AST: Aspartate aminotransferase
ALT: Alanine aminotransferase
cACLD: Compensated advanced chronic liver disease
CI: Cardiac index
CSPH: Clinically significant portal hypertension
CVP: Central venous pressure
EUS: Endoscopic ultrasound
EUS-PPG: Endoscopic ultrasound-guided portal pressure gradient
FHVP: Free hepatic venous pressure
HVPG: Hepatic venous pressure gradient
LSM: Liver stiffness measurement
MAFLD: Metabolic-associated fatty liver disease
MRE: Magnetic resonance elastography
NSBBs: Non-selective beta-blockers
PH: Portal hypertension
PVP: Portal venous pressure
SHAPE: Subharmonic-aided pressure estimation
SSM: Splenic stiffness measurement
tDT: Terminal thoracic duct
TE: Transient elastography
RTE: Real-time tissue elastography
WHVP: Wedged hepatic venous pressure
